# Vaccine Certificates Must Go Digital: An Urgent Call for Better Public Health Outcomes

**DOI:** 10.2196/65740

**Published:** 2024-11-26

**Authors:** Jarbas Barbosa da Silva Júnior, Sebastian Garcia-Saiso, Myrna Marti, Daniel Salas, Marcela Contreras, Martha Velandia-Gonzalez, Daniel Luna, Jennifer Nelson, James Fitzgerald, Ernesto Bascolo, Ivy Lorena Talavera Romero, May Chomali, Walter H Curioso, Fernando Plazzotta, Carlos Otero, Alejandro Lopez Osornio, Tessa Lennemann, Karen Salinas, Marcelo D'Agostino

**Affiliations:** 1 Pan American Health Organization, 525 23rd ST NW, Washington, DC, 20037, United States, 1 7034737961; 2 Department of Public Health Informatics, Hospital Italiano de Buenos Aires, Buenos Aires, Argentina; 3 Social Sector and Health Division, Inter American Development Bank, Washington, DC, United States; 4 Centro Nacional de Sistemas de Informacion en Salud - CENS, Santiago de Chile, Chile; 5 Universidad Continental, Lima, Peru; 6 Institute for Clinical Effectiveness and Health Policy, Buenos Aires, Argentina; 7 Department of Digital Innovation for Pandemic Control (DIPC), German Agency for International Cooperation, Berlin, Germany

**Keywords:** medical informatics, health systems, immunizations, public health, viewpoint, health challenges, digital vaccine certificates, outbreak management, outbreak, accuracy, healthcare, surveillance, health outcomes, global health, mobile phone

## Abstract

From our roles within international public health organizations, we have collectively witnessed the global challenges presented by outdated health information systems, platforms, and applications. The COVID-19 pandemic has clearly exposed the limitations of our current paper-based vaccine certification methods and highlighted the deficiencies of outdated technological platforms that lack interoperability standards, a situation that underscores the critical need for a digital transformation in how we manage and verify immunization records. Digital vaccination certificates are understood to be secure, electronically stored, and easily accessible records that provide verifiable proof of a person’s immunization status. The Pan American Health Organization (PAHO) envisions leveraging digital technologies to strengthen health systems, enhance data-driven decision-making, and improve health outcomes. The organization’s vision emphasizes the integration of innovative technologies to build resilient and responsive health systems capable of addressing modern public health challenges. In an era of unprecedented technological advancement, our continued reliance on paper-based vaccine certificates is not just anachronistic—it is a significant liability for global public health that impacts the efficiency and effectiveness of our health systems on multiple fronts, limiting our ability to respond to public health crises effectively. With the strategic guidance from its Member States, PAHO has agreed to move toward the digital transformation of the health sector across the entire continent with an initiative that aims to improve health outcomes, ensure equitable access to health services, and enhance the overall efficiency of health systems in the Americas. The roadmap for this digital transformation outlines strategic actions and goals to achieve a connected, efficient, and resilient health sector.

## Introduction

From our roles within international public health organizations, we have collectively witnessed the global challenges presented by outdated health information systems, platforms, and applications. The COVID-19 pandemic has clearly exposed the limitations of our current paper-based vaccine certification methods and highlighted the deficiencies of outdated technological platforms that lack interoperability standards, a situation that underscores the critical need for a digital transformation in how we manage and verify immunization records. Digital vaccination certificates are understood to be secure, electronically stored, and easily accessible records that provide verifiable proof of a person’s immunization status. The Pan American Health Organization (PAHO) envisions leveraging digital technologies to strengthen health systems, enhance data-driven decision-making, and improve health outcomes. The organization’s vision emphasizes the integration of innovative technologies to build resilient and responsive health systems capable of addressing modern public health challenges [1]. In an era of unprecedented technological advancement, our continued reliance on paper-based vaccine certificates is not just anachronistic—it is a significant liability for global public health that impacts the efficiency and effectiveness of our health systems on multiple fronts, limiting our ability to respond to public health crises effectively [2]. With the strategic guidance from its Member States, PAHO has agreed to move toward the digital transformation of the health sector across the entire continent with an initiative that aims to improve health outcomes, ensure equitable access to health services, and enhance the overall efficiency of health systems in the Americas. The roadmap for this digital transformation outlines strategic actions and goals to achieve a connected, efficient, and resilient health sector [3].

## The Shortcomings of Paper Certificates

A vaccination certificate is a health document used to track the vaccines an individual has received. It is kept in the household, in most countries in paper format, by the individual or their caregiver. It is designed to be integrated into the health information system and complements other health records maintained by health facilities. Home-based records have a long history, and their content has been expanded over time, leading to changes in their use. According to World Health Organization (WHO), although home-based records have been widely implemented for decades, evidence of their benefits and challenges has not been systematically reviewed and summarized. For health care providers and health care service delivery, the potential benefits of home-based records include access to important health data about women, newborns, and children; better risk detection and prompt referral; improved communications with caregivers; and strengthened links among different health workers and services [4]. For women, caregivers of children and family members, home-based records can facilitate learning and awareness of health problems, promote detection of risks and timely action, encourage continuity of care, and encourage positive health behaviors during pregnancy, among others [5]. However, paper certificates present some challenges like (1) obstructing rapid public health responses, particularly in emergency situations where the need for quick verification of vaccination status is critical; (2) being vulnerable to errors, illegibility, delays in verification, in addition to the lack of care or loss of certificates, complicating efforts to assess population immunity and deploy targeted interventions promptly; (3) being restricted to effective public health surveillance and monitoring due the lack of real-time data from paper certificates; and (4) difficulties to establish the actual vaccination coverage by neighborhoods and districts among others.

Health authorities should rely on accurate and timely data to monitor vaccination coverage, identify gaps and barriers, and predict outbreaks. Paper certificates do not support the rapid collection and analysis of vaccination data, limiting our ability to implement proactive public health measures. Time-consuming verification processes slow down operations, diverting valuable resources away from patient care and public health initiatives that increase the burden on health systems is particularly problematic in identifying undervaccinated communities and the barriers they are facing to get the vaccines and during vaccination campaigns and travel, where quick and reliable verification is essential [4,5]. In the particular case of migration or traveling, paper certificates complicate cross-border public health efforts, as highlighted by the current migration crisis in the region [6,7] where inconsistencies in record-keeping and difficulties in verifying paper-based vaccination status impede international collaboration and the implementation of coordinated public health strategies. This limitation is especially critical in managing global health threats that require a unified response.

The implementation of digital certificates for all vaccines used in national immunization programs “democratizes” access to information through semantic interoperability standards that leave behind former barriers like language, specific countries‘ vaccination models and schemes, and incompatibilities of commercial schemes (combinations, specific commercial brands, etc).

## Digital Vaccine Certificates: A Public Health Imperative

Digital vaccine certificates offer a transformative solution to these challenges, significantly enhancing public health capabilities. One of the most crucial benefits is the ability to provide real-time data for public health surveillance. Digital certificates enable health authorities to continuously monitor vaccination in the target populations, identify underserved populations, and respond quickly to emerging threats. With proper consent, aggregated data can provide valuable insights for understanding vaccination patterns and effectiveness. This real-time insight is vital for effective outbreak management and prevention. Enhanced accuracy and security are primary benefits, as digital certificates reduce the risk of errors, falsification, and unauthorized access. With centralized and interoperable databases and automatic updates, digital certificates ensure that vaccination records are accurate and up-to-date, supporting reliable public health interventions [8].

Several countries and regions have already successfully implemented digital vaccine certificate systems, demonstrating the practical benefits of these innovations. The European Union’s Digital COVID Certificate, for example, has been pivotal in facilitating cross-border travel across member states by providing a standardized, secure method of verifying vaccination status. This has not only streamlined travel procedures but also enhanced public health monitoring across the region. In Latin America and the Caribbean, initiatives such as Latin America and Caribbean interoperability initiative to facilitate the PASSing of digital health records across borders (LACPASS) and the Digital Documentation of COVID-19 Certificates, supported by PAHO and the Inter-American Development Bank, have provided a standardized platform for cross-border verification of COVID-19 vaccination status. These programs have not only improved cross-border travel but also ensured secure and verifiable vaccination records, fostering regional cooperation and enhancing public health responses. These case studies illustrate the tangible benefits of digital vaccine certificates, not only in enhancing public health responses but also in supporting broader societal and economic functions during and beyond the COVID-19 pandemic.

Digital certificates also promote data-driven public health strategies. Real-time data collection and analysis enable health authorities to track vaccination progress, identify gaps, and allocate resources efficiently. This capability is crucial for maintaining high vaccination coverage and preventing outbreaks, ultimately enhancing public health resilience.

From a global health perspective, digital certificates facilitate better coordination and collaboration. Interoperability across health systems and jurisdictions in and between countries allows for the easy and secure sharing of vaccination records, supporting unified public health responses [9]. This interoperability is essential for managing cross-border health threats and ensuring global health security. Recent efforts, such as the 2024 amendments to the 2005 International Health Regulations to include both paper and digital health certificates, and the WHO Standards-based, Machine-readable, Adaptive, Requirements-based, and Testable (SMART) Guidelines help to establish international policy and standards to enable interoperable digital vaccines globally [10]. The accessibility of digital certificates also supports equitable public health outcomes [11]. Open and real-time access via smartphones or secure online portals, and, digital certificates ensure that vaccination status is easily verifiable, reducing barriers to health care and public services. This accessibility is particularly important for vulnerable populations, during travel or migration, and to access education or permission to work, where proof of vaccination is often required. Another important aspect relates to patient empowerment, as they gain greater control over their vaccination records, in line with patient-centered health care trends.

## Enhancing Primary Health Care

The digitalization of vaccine certificates supports the strengthening of primary health care by improving the continuity of care and facilitating health care providers with immediate access to a patient’s vaccination history, which enables more informed decision-making and timely interventions. In addition, digital certificates facilitate better coordination among various levels of the health system [12], which enhances patient care, reduces the risk of missed vaccinations, increases quality, and supports comprehensive health management. Primary health care providers can easily share vaccination data with specialists, hospitals, and public health authorities, ensuring that all stakeholders are informed and aligned in their efforts to maintain high vaccination coverage and protect the population against vaccine-preventable diseases. This coordination is essential for addressing public health challenges and achieving broader health system goals [13]

It also allows not only the knowledge of the immunity coverage of people, but also the creation of professional profiles, with metadata necessary for the care of health personnel, such as, the necessary antibody response. In this sense, digital certificates allow the creation of information flows by profiles with much more reliable information.

## Connecting to the Global Digital Health Certification Network

The WHO’s Global Digital Health Certification Network (GDHCN) represents a significant step forward in establishing a robust digital public health infrastructure. This open-source platform designed to support the bilateral verification of health documents, including vaccination certificates, across borders can help health authorities to ensure the authenticity of digital records, enhance interoperability, and facilitate global public health responses. The Global Digital Health Certification Network builds upon existing regional networks and integrates with various digital health systems, making it a critical component in the global digital transformation of health records [11].

## Addressing Concerns

As we advocate for the transition to digital vaccine certificates, it is crucial to address valid concerns regarding data privacy, security, and the protection of vulnerable groups. Implementing robust data protection policies and measures, and adhering to international standards, is essential to safeguard individuals’ personal information. Ensuring that digital certificates are secure and that privacy is respected will build public trust and encourage widespread adoption. Equity and accessibility are critical considerations. Solutions must be developed for individuals without access to smartphones, the internet, or even electricity, to ensure that no one is left behind [14-16]. For instance, family records should be considered, as minors do not, and should not, manage their own records; thus, their caregiver should be able to do so. In addition, despite the high penetration of devices in the region, some families or groups still rely on a single cell phone (as observed during the pandemic). Therefore, applications must allow for managing multiple accounts on one device. Providing alternative options and ensuring equitable access to digital certificates is vital for an inclusive approach. Ethical considerations are also crucial: who has access to the certificates? Who grants access to my certificates? Who needs permission (eg, the private sector or an employer) and who does not (eg, the government or all governments)? It is also important to emphasize that, in terms of interoperability, integration with national and regional information systems should be considered. In addition, integration with clinical records in general, including those in the private sector, is essential. Technical reliability is another concern that must be addressed. Developing robust infrastructure, info-structure, and user-friendly interfaces will be crucial for the successful adoption of digital certificates. Ensuring that the technology is reliable and accessible will prevent technical issues and enhance user experience. Finally, international standardization is necessary for the interoperability of digital certificates. Collaboration between nations and international health bodies is essential to develop and implement interoperable standards that facilitate the easy sharing of vaccination records across borders and within countries [17,18].

## Implementation Strategy

Based on our collective regional and global experience, we propose a comprehensive implementation approach, including (1) pilot programs in diverse settings, which will help identify and address potential issues, providing valuable insights for scaling up the initiative; (2) investment in digital infrastructure, particularly in underserved areas, which is crucial to ensure that all regions benefit from this transition; (3) comprehensive public education campaigns, which will raise awareness about the benefits of digital certificates and address concerns regarding privacy and security; (4) training programs for health care providers and administrators, which will equip them with the necessary skills to manage and verify digital certificates effectively; (5) maintaining legacy systems during the transition period to ensure continuity and provide a safety net while the new digital systems are being implemented; (6) updating legal and regulatory frameworks to recognize digital certificates and provide a clear legal basis for their use; (7) developing policies to ensure equitable access to digital systems and to address the needs of marginalized and underserved populations, ensuring that everyone can benefit from the transition to digital certificates; and (8) fostering global cooperation through partnerships between nations to share best practices, lessons learned, and technical expertise in implementing digital certificate systems to accelerate adoption and ensure interoperability across borders.

## A Call to Action

The need for this transition is immediate. As we continue moving toward more resilient health systems, digital vaccine certificates are not merely a technological upgrade—they are a critical tool for global health security. They can enhance our ability to prevent outbreaks, manage public health risks, and facilitate safe international travel and commerce. Furthermore, digital certificates align with the broader movement toward digital health systems, supporting the UN Sustainable Development Goals, particularly SDG 3 (Good Health and Well-being) and SDG 9 (Industry, Innovation, and Infrastructure).

A key driver for this transformation will be the Pan-American Highway for Digital Health (PH4H) initiative. Led by the Inter-American Development Bank, in collaboration and coordination with the PAHO, PH4H aims to enable connected health for all in Latin America and the Caribbean. This initiative is designed to facilitate secure, efficient, and interoperable health data exchange both within and between countries, allowing people to share and access their health information effectively. PH4H aims to provide patients with better health care services, regardless of their location, and enhance care for those who move temporarily for work or study, as well as for migrants. Highlighted at the indicator 2.1.4 of the Plan of Action for Strengthening Information Systems for Health 2024‐2030, the digital highway will enable countries to be better prepared for future pandemics and other health threats by strengthening public health surveillance, optimizing access to limited human resources, promoting research and innovation, and leading to more efficient public health policies and boosting regional economies [19].

During the recent COVID-19 pandemic, we have seen the tremendous impact that interconnected and interoperable information systems and innovative digital health solutions can have on communities worldwide. Digital vaccine certificates represent a crucial next step in our global health infrastructure and this transition from paper to digital will require collaboration between health organizations, governments, and technology experts. It will demand investment, both financial and intellectual, but the returns—in terms of lives saved, pandemics averted, and a more resilient global health system—are immeasurable. Countries recommitted to primary health care through the Astana Declaration in October 2018, marking a renewal of the Alma Ata Declaration, emphasizing universal health coverage and highlighting digital technologies as a key strategy [20]. Also, endorsed by the World Health Assembly, WHO launched the Global Strategy on Digital Health to support home and community care, clinical decision-making, provider education, and health information systems. The vision of the Global Strategy is to improve health for everyone by accelerating the development and adoption of scalable, sustainable, and person-centric digital health solutions [21] whose transformative potential was underscored by the COVID-19 pandemic. However, achieving large-scale implementation in primary health care settings globally requires overcoming significant policy and operational challenges [13].

We call upon our colleagues in the global health community, policy makers, and technology leaders to join us in this crucial step. The continued use of paper-based vaccine certificates is no longer acceptable in the face of modern public health demands and the ongoing priorities assigned to the digital transformation of governments as a whole. Adopting digital vaccine certificates, we can enhance the effectiveness of vaccination programs, improve public health outcomes, and ensure that no one is left behind in the global effort to combat not only infectious diseases, but also other pressing global health issues. As we move forward with this digital transformation, we must ensure equitable access and digital inclusion, leaving no one behind in our global health efforts. The time for digital vaccine certificates is now.

## Abbreviations

GDHCN: Global Digital Health Certification Network
LACPASS: Latin America and Caribbean interoperability initiative to facilitate the PASSing of digital health records across borders
PAHO: Pan American Health Organization
PH4H: Pan-American Highway for Digital Health
SMART: Standards-based, Machine-readable, Adaptive, Requirements-based, and Testable
UN SDG: United Nations Sustainable Development Goals
WHO: World Health Organization
